# 

**Published:** 1891-06

**Authors:** 


*Cases for binding, price 7d. each, may be had of the Publisher,*

J. W. ARROWSMITH, *Quay Street, Bristol.*

### *PUBLICATIONS RECEIVED.*

From Ev. E. Carreras, St. Louis:
*The Alienist and Neurologist.* January, 1891.

From Cassell & Company Limited, London:
*Materia Medica and Therapeutics.* By J. Mitchell Bruce, M.D. 1891.

From *EL CENSOR*, Buenos Ayres:
*Boletin de Sanidad Militar.* No. 2. February 1, 1891.
*Anales de la Asistencia Pública.* Nos. 4 to 6. 1891.

From J. & A. Churchill, London:
*Proclivity of Women to Cancer.* By Herbert Snow, M.D. 1891.

From F. A. Davis, Philadelphia:
*Medical Symbolism.* By Thomas S. Sozinskey, M.D. 1891.
*Fever.* By Hobart Amory Haré, M.D. 1891.
*Materia Medica and Therapeutics.* Vol. II. By John V. Shoemaker, M.D. 1891.

From Harrison & Sons, London:
*On the Physiology of Asphyxia, and on the Anæsthetic Action of Pure Nitrogen.* By George Johnson, M.D. [1891.] Reprint.

From J. N. Klingelfuss y Ca. Buenos Aires:
*Anales del Departmento Nacional de Higiene.* Nos. 1, 2. 1891.

From Ledger, Smith, & Co., London:
*The Medical Digest.* By Richard Neale, M.D. Third Edition. 1891.

From H. K. Lewis, London:
*Comparative Climatology.* By Bertram Thornton. 1891.
*Diseases of the Nose and Throat.* By Procter S. Hutchinson. 1891.
*Elements of Medicine.* By Alfred H. Carter, M.D. Sixth Edition. 1891.
*The British Journal of Dermatologv.* January—June, 1891.
*Lewis's Medical Vocabulary.* Second Edition. 1891
*Notes on Additions to the British Pharmacopœia.* By Frederick T. Roberts, M.D. 1891.
*Intubation of the Larynx.* By James B. Ball, M.D. 1891.
*New Official Remedies, B.P.* 1890. By A. W. Gerrard. 1891.

From Longmans, Green, & Co., London:
*Varicocele.* By William H. Bennett. 1891.

From Alex. Macdougall, Glasgow:
*Medico-Chirurgical Society of Glasgow. Discussion on Anæsthetics.* Edited by J. Walker Downie, M.B. 1891.

From Oliver & Boyd, Edinburgh:
*Notes on Surgery for Nurses.* By Joseph Bell, M.D. Third Edition. 1891.
*Introduction to the Diseases of Infancy.* By J. W. Ballantyne, M.D. 1891.

From The Open Court Publishing Co., Chicago:
*The Monist.* April, 1891.

From L. & J. Parnell, London:
*Rhyming and Mnemonic Key to Materia Medica.* By M.D. (n.d.)

From Young J. Pentland, Edinburgh:
*Pulmonary Tuberculosis.* By. R. W. Philip, M.D. 1891.
*Diseases of the Digestive Organs in Children.* By Louis Starr, M.D. Second Edition. 1891.

From John Morgan Richards, London:
*Medical Reprints.* March—May, 1891.

From Walter Scott, London:
*Bacteria and their Products.* By German Sims Woodhead, M.D. 1891.

From 126 East 29th Street, New York:
*Twenty-third Annual Report of the New York Orthopædic Dispensary.* 1891.

